# Molecular differences in susceptibility of the kidney to sepsis-induced kidney injury

**DOI:** 10.1186/s12882-017-0602-x

**Published:** 2017-05-31

**Authors:** Martin Matejovic, Lenka Valesova, Jan Benes, Roman Sykora, Roman Hrstka, Jiri Chvojka

**Affiliations:** 1 1st Medical Department, Faculty of Medicine in Pilsen, Charles University, Plzen, Czech Republic; 2 Experimental Intensive Care Unit, Biomedical Centre, Faculty of Medicine in Plzen, Charles University, alej Svobody 1655/76, Plzen, Czech Republic; 3 Regional Centre for Applied Molecular Oncology, Masaryk Memorial Cancer Institute, Zluty kopec 7, Brno, Czech Republic; 4 Department of Anesthesia and Intensive Care Medicine, Faculty of Medicine in Pilsen, Charles University, Plzen, Czech Republic; 5 1st Medical Department, Teaching Hospital, alej Svobody 80, 304 60, Plzen, Czech Republic

**Keywords:** Sepsis, Acute kidney injury, Animal models, Gene expression

## Abstract

**Background:**

Septic acute kidney injury affects 40–50% of all septic patients. Molecular differences between septic patients with and without acute kidney injury (AKI) are only poorly understood. Here, we investigated gene expression changes that differentiated the subjects who developed septic AKI from those who did not and coupled this approach with traditional parameters of renal physiology.

**Methods:**

In 15 anesthetized, mechanically ventilated and instrumented pigs, progressive sepsis was induced either by peritonitis or by continuous intravenous infusion of *Pseudomonas aeruginosa*. Animals received standard intensive care including goal-directed hemodynamic management. Analyses were performed on kidneys from sham operated animals, septic pigs without AKI, and pigs with septic AKI. Before, and at 12, 18 and 22 h of progressive sepsis, systemic and renal hemodynamics, cortex microcirculation and plasma IL-6 and TNF-α were measured. At 22 h whole kidney expression of pre-selected genes was analyzed by quantitative Real Time PCR.

**Results:**

Animals with septic AKI had systemic hemodynamic phenotype (normo- or hyperdynamic) comparable with non-AKI subjects, but demonstrated higher plasma levels of cytokines, an increase in renal vascular resistance and early fall in cortical microcirculatory blood flow. The genes whose expression discriminated septic AKI from non-AKI included Toll like receptor 4 (up-regulated 2.7-fold, *P* = 0.04); Cyclooxygenase-2 (up-regulated 14.6-fold, *P* = 0.01), Angiotensin II Receptor (up-regulated 8.1-fold, *P* = 0.01), Caspase 3 (up-regulated 5.1-fold, *P* = 0.02), Peroxisome Proliferator-Activated Receptor Gamma, Coactivator 1 Alpha (down-regulated 2-fold, *P* = 0.02).

**Conclusions:**

In this preliminary experimental study, kidney gene expression was profoundly different in animals that developed septic AKI as opposed to septic animals that did not. The biological functions of the genes differentially expressed support a role of inflammatory overstimulation coupled with metabolic and apoptotic molecular responses in early septic AKI. Cyclooxygenase-2 and angiotensin type 2 receptor-dependent downstream mechanisms appear fruitful targets for future mechanistic research.

## Background

Sepsis is the most common cause of acute kidney injury (AKI) [1]. The expanding population of patients with sepsis and AKI, and the associated excess in mortality provide strong basis for basic research aimed at elucidating mechanisms underlying their complex pathophysiology. Both hemodynamic and non-hemodynamic pathogenic pathways have been shown to contribute to the development of septic AKI (S-AKI) [2, 3]. However, a more comprehensive understanding of the renal pathobiology in sepsis requires technologies of molecular biology. Unfortunately, several factors make the human research into cellular and molecular biology in critically ill patients problematic. In particular, difficult access to the human renal tissue and several confounding factors including disease state, the patient’s comorbidities and treatment history represent main impediments to the study of human S-AKI. Therefore, the use of clinically and biologically relevant animal models remains a cornerstone in the sepsis and AKI research. We have established large animal models of sepsis that allowed us to generate two distinct groups of septic animals, those with and without AKI [3]. The separation of animals to those with and without S-AKI is a unique tool to isolate and study factors discriminating AKI from non-AKI. Recently, we reported that early abnormal host response coupled with subsequent renal vasoconstriction despite apparent sepsis-induced systemic vasodilatation were major discriminating factors associated with the development of S-AKI in porcine models [3]. Herein, we extend our previous work and utilize genomic tools to identify differences in renal molecular patterns between those two distinct groups. Rather than utilizing genome-wide approach, we prospectively selected subset of genes most likely to be implicated in the key pathogenic pathways. These can be broadly clustered into the following major functional categories – inflammation/danger response (Toll like receptor 2, Toll like receptor 4, Tumor necrosis factor alpha), mitochondrial energy metabolism (Peroxisome Proliferator-Activated Receptor Gamma, Coactivator 1 Alpha); cellular stress response and vasoregulation (Heat shock protein HSP 90, Heat shock protein HSP 70; Heat shock protein HSP 27, Heat shock cognate 70 kDa protein, Hypoxia inducible factor 1, Inducible nitric oxide synthase, Cyclooxygenase-2, Angiotensin II Receptor, Type 2), and apoptosis (BCL2-Associated X Protein, Caspase 3).

## Methods

From the set of experimental animals reported in the previous study [3], we have investigated 15 pigs, in which kidney tissue samples were available for further analyses: five sham- operated, time-, age- and weight-matched controls (control group), five septic animals without AKI (non-AKI group) and five animals developing septic AKI (AKI group). Thus, the present approach is distinct, but complementary to our previous work [3].

### Anesthesia and surgical preparation

Anesthesia and surgical instrumentation have been described in detail in the previous report [3]. Briefly, anesthesia was induced with atropine, propofol and ketamine. After endotracheal intubation, anesthesia, analgesia and muscle paralysis were achieved with thiopental, fentanyl and pancuronium. Ventilator settings for mechanical ventilation were: fraction of inspired O_2_ (FiO_2_) 0.4, positive end-expiratory pressure (PEEP) 6 cmH_2_O, tidal volume, 8–10 mL·kg^−1^, respiratory rate was adjusted to maintain normocapnia. A fibre-optic arterial catheter was inserted into the femoral artery for continuous blood pressure measurement, intermittent double-indicator transpulmonary dilution (COLD Z-021, PULSION Medical Systems GmbH, Germany) and blood sampling. Central venous and pulmonary artery catheters were introduced via jugular veins. A midline laparotomy was performed and a precalibrated ultrasound flowprobe (Transonic Systems, Ithaca, NY) was placed around the left renal artery. Laser Doppler probe (PF 404, Suturable angled probe, Perimed, Jarfalla, Sweden) was placed directly over the renal cortex for cortical microcirculation assessment and a double-lumen catheter was inserted into the left renal vein for renal venous pressure measurements and blood sampling. Peritoneal drainage was inserted before abdominal wall closure and epicystostomy was performed under ultrasound control. A recovery period of 6 h was provided before the baseline measurement.

### Measurements and calculations

Measurements and calculations performed at each time-point (baseline, 12,18 and 22 h after induction of sepsis) have been described in detail in the previous report and comprise systemic and renal hemodynamics, O_2_ exchange, blood gas analyses, plasma creatinine, leukocyte and platelet counts, tumor necrosis factor alpha (TNF-α) and interleukin 6 (IL-6). To correct for dilution effects resulting from volume resuscitation IL-6 and TNF-α levels were normalized for plasma protein content. AKI was defined according to the AKIN criteria as an increase of more than 26.4 μmol/l or 150% in serum creatinine from baseline.

### Gene expression analysis by Real-Time PCR

At the time of animal sacrifice, left kidney was rapidly excised and pyramid-shaped kidney specimens were taken, washed with sterile saline, flash-frozen in liquid nitrogen and kept at −80 °C until RNA purification. RNA was extracted using the RNeasy Isolation Kit (Qiagen) according to the manufacturer’s instructions. RNA concentration and purity were controlled by UV spectrophotometry (A260:A280 > 2.0; A260:A230 > 1.8). RNA integrity was checked using Agilent 2100 Bioanalyzer and only non-degraded RNA characterized by RIN (RNA Integrity Number) higher than 7 with no DNA contamination signs was processed. cDNA synthesis was carried out using the M-MLV reverse transcriptase (Invitrogen). Toll like receptor 2 (TLR2, Assay ID: Ss03381278_u1), Toll like receptor 4 (TLR4, Assay ID: Ss03389779_m1), Tumor necrosis factor alpha (TNF-α, Assay ID:Ss03391316_g1); Peroxisome Proliferator-Activated Receptor Gamma, Coactivator 1 Alpha (PGC-1 alpha, Assay ID:Ss03393114_u1); Heat shock protein HSP 90 (HSP90AA1, Assay ID:Ss03391152_g1), Heat shock protein HSP 70 (HSP70, Assay ID:Ss03374255_m1); Heat shock protein HSP 27 (HSP27, Assay ID:Ss03378829_u1), Heat shock cognate 70 kDa protein (HSPA5, Assay ID:Ss03374255_m1), Hypoxia inducible factor 1 (HIF1A, Assay ID:Ss03390447_m1), Inducible nitric oxide synthase (NOS2, Assay ID:Ss03374608_u1), Cyclooxygenase-2 (PGHS-2, Assay ID:Ss03394695_g1), Angiotensin II Receptor, Type 2 (AGTR2, Assay ID:Ss03376937_u1), BCL2-Associated X Protein (BAX, Assay ID:Ss03375842_u1) and Caspase 3 (CASP3, Assay ID:Ss03382792_u1)) were determined using TaqMan Expression Assays (all Applied Biosystems). Obtained expression data were normalized according to the expression of GAPDH and 18S rRNA (both Applied Biosystems) with similar results.

### Experimental protocols and supportive treatment

Severe sepsis was induced either by continuous intravenous infusion of Pseudomonas aeruginosa (1 × 10^9^ colony-forming units/mL, *n* = 3 in non-AKI group, *n* = 2 in AKI group) or fecal peritonitis (*n* = 2 in non-AKI group, *n* = 3 in AKI group) as described previously [3]. The infusion rate of Pseudomonas aeruginosa was titrated to clinical goal of moderate pulmonary hypertension (MPAP 35-40 mmHg). In the peritonitis group, sepsis was induced by inoculating 0.5 g/kg of autologous feces incubated in 200 ml saline for 8 h at 37 °C through the drains into the abdomen. Continuous infusion of balanced crystalloids (Plasmalyte, Baxter Healthcare, Deerfield, IL, United States or Ringerfundin, BBraun Melsungen Ag, Melsungen, Germany) were used as a fluid replacement in dose 15 ml/kg/h during the surgery and reduced to 7 ml/kg/h thereafter. In addition to crystalloid solution, 6% hydroxyethyl starch 130 kD/0.4 (Voluven 6%, Fresenius Kabi Deutschland GmbH, Bad Homburg, Germany) was infused to maintain normovolemia in a goal-directed fashion guided by filling pressures response and ITBV measurement. Continuous i.v. noradrenaline was administered if mean arterial pressure (MAP) fell below 65 mmHg and titrated to maintain MAP above 70 mmHg. When the last set of data had been obtained, the animals were euthanized by potassium chloride injection under deep anesthesia and section was performed.

### Statistical analysis

All data are presented as median (quartiles) unless otherwise stated. The calculations were done using SigmaStat software version 3.5 (Systat Software Inc., Erkrath, Germany). After exclusion of normality using the Kolmogorov– Smirnov test, differences within each group and between the groups were analyzed with the Kruskal-Wallis rank sum test for multiple comparisons and a subsequent Dunn’s test. A *p* value of less than 0.05 was regarded as statistically significant.

## Results

### Systemic hemodynamics and inflammatory response

The systemic hemodynamic and inflammatory responses to the infectious challenge in pigs with and without AKI are summarized in Table 1. There were no statistically significant differences in any measured variables between NON-AKI and AKI pigs at baseline. Cardiac output was well maintained in both groups, and even increased in NON-AKI animals by the end of the experiment. Systemic vascular resistance progressively and comparably dropped in both groups, without any intergroup differences. Total dose of noradrenalin was 0.29 (0.19–1.08) vs. 0.15 (0.08–0.20) mg/kg; *p* = 0.15 in the AKI and the NON-AKI group, respectively. Likewise, total amount of fluids did not significantly differ between AKI and NON-AKI animals (data not shown). Both groups showed significant systemic inflammatory response as evidenced by the course of TNF-alfa and IL-6 plasma levels, although there was a clear-cut tendency of these markers to increase more markedly in the AKI group.

**Table 1:** Parameters of systemic hemodynamics and inflammation

	Baseline	12 h	18 h	22 h
MAP [mmHg]				
NON-AKI	93 [86–111]	80 [72–91]	76 [73–86]	78 [70–89]
AKI	99 [97–114]	74 [71–101]	70 [65–98]	62 [57–78]*
CO [ml/kg/min]				
NON-AKI	90 [73–97]	102 [64–117]	105 [82–122]	159 [97–168]*
AKI	99 [90–108]	139 [100–202]	173 [133–185]	94 [72–176]
SVR [dyne.s.cm^−5^]				
NON-AKI	2411 [1822–2844]	1973 [1401–2059]*	1382 [1246–1453]*	778 [583–1765]*
AKI	2206 [1998–2821]	1463 [707–1824]*	720 [619–1572]*	854 [610–1453]*
ITBVI [ml/kg]				
NON-AKI	23 [21–28]	27 [22–30]	22 [21–26]	23 [22–26]
AKI	23 [22–25]	25 [21–31]	27 [23–29]	24 [20–27]
TNF alfa [pg/g of protein]				
NON-AKI	1.3 [0.8–1.5]	4.4 [2.4–5.3]*	10 [8.9–19.7]*	14.5 [5.7–23.2]*
AKI	1.4 [1.4–3.5]	20.6 [10.0–24.3]*	23.4 [6.0–33.8]*	22.7 [5.3–133.5]*
IL-6 [pg/g of protein]				
NON-AKI	4.0 [3.0–5.5]	5.9 [5.1–11.9]	33.0 [24.3–119.3]*	13.2[6.9–543.9]*
AKI	2.1 [1.1–6.4]	217 [164–479]#*	474 [306–1016]#*	384 [331–9309]*

Legend:

*p < 0.05 vs. Baseline (RM ANOVA on Ranks; Dunn’s method of multiple comparisons)

#*p* < 0.05 AKI vs. AKI-FREE (ANOVA on Ranks; Dunn’s method of multiple comparisons)

### Renal response to sepsis in pigs with and without AKI

Serum creatinine levels increased significantly in AKI animals, while no changes were observed in the NON-AKI group (Table 2). Both renal blood flow and renal vascular resistance remained unaffected in NON-AKI animals. By contrast, gradually increased renal vascular resistance was observed in AKI group and was accompanied by reduced renal blood flow (Table 2). Compared to baseline values, the renal cortical microcirculatory blood flow fell early only in the AKI group (Table 2).

**Table 2:** Renal effects of sepsis in pigs with and without AKI

	Baseline	12 h	18 h	22 h
Creatinine [umol/l]				
NON-AKI	99 [91–118]	95 [79–115]	98 [85–113]	101 [93–119]
AKI	103 [97–110]	111 [94–131]	136 [117–164]*#	163 [137–217]*#
Renal Blood Flow [ml/min/kg]				
NON-AKI	6 [5–6]	4 [3–7]	5 [3–7]	4 [3–5]
AKI	5 [4–8]	3 [2–4]	3 [1–5]*	1 [0.5–2]*#
Renal Vascular Resistance [mmHg/l/min]				
NON-AKI	422 [345–494]	385 [314–461]	320 [284–417]	417 [358–465]
AKI	419 [321–637]	782 [537–1003]	1457 [567–2374]	2433 [1400–3886]*#
Renal cortical microcirculation (Laser Doppler Flowmetry) [% of baseline value]				
NON-AKI	100 [100–100]	108 [66–116]	100 [77–123]	84 [52–110]
AKI	100 [100–100]	50 [28–61]*	50 [38–56]*	40 [27–57]*#

Legend:

*p < 0.05 vs. Baseline (RM ANOVA on Ranks; Dunn’s method of multiple comparisons)

#*p* < 0.05 AKI vs. AKI-FREE(ANOVA on Ranks; Dunn’s method of multiple comparisons)

### Kidney tissue expression of selected genes

**Fig. 1:**
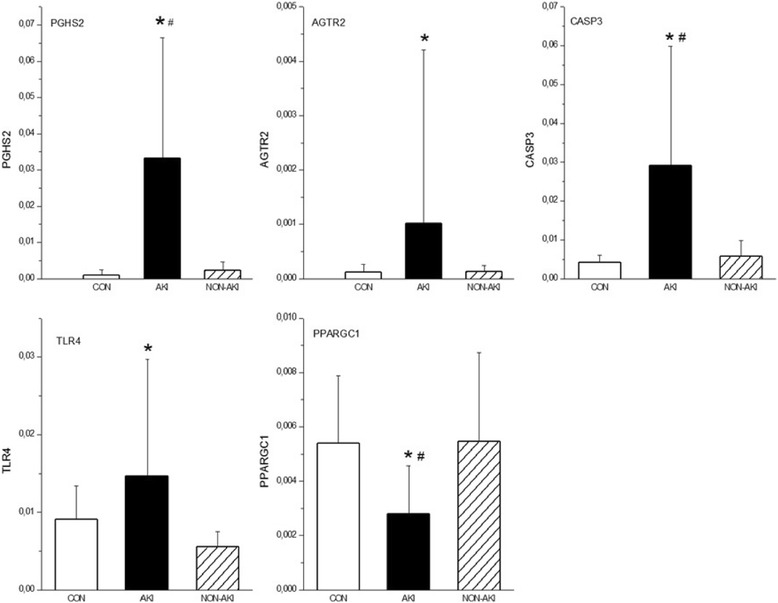
Whole kidney tissue mRNA levels of genes whose expression significantly changed at 22 h in septic animals developing AKI (AKI) compared to septic animals without AKI (NON-AKI) and sham-operated control groups (CON). Results are expressed as mean ± SEM. ★ designates *p* < .05 vs. NON-AKI. # designates *p* < .05 vs. control. PGHS-2 - Cyclooxygenase-2, AGTR2 - Angiotensin II Receptor, Type 2, CASP3 - Caspase 3, TLR4 - Toll like receptor 4, PPARGC1 - Peroxisome Proliferator-Activated Receptor Gamma, Coactivator 1 Alpha

**Fig. 2:**
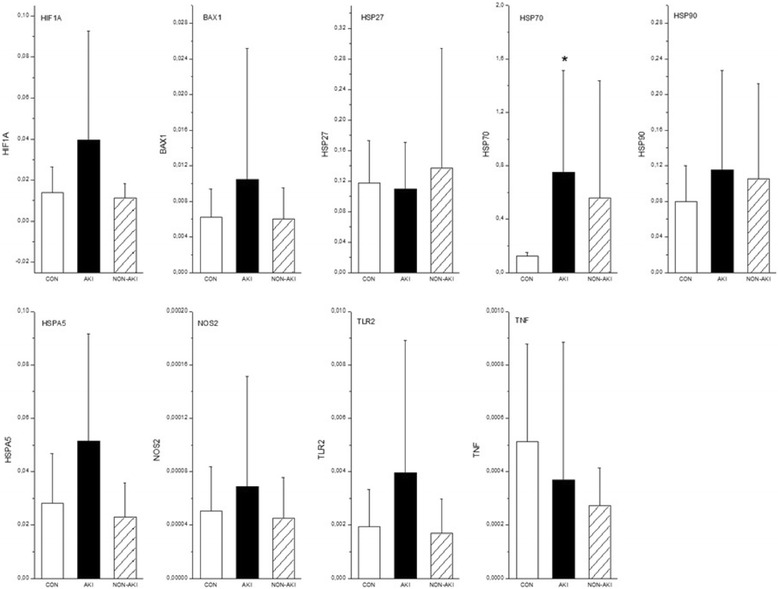
Whole kidney tissue mRNA levels of genes whose expression did not change in septic animals developing AKI (AKI) compared to septic animals without AKI (NON-AKI) in septic animals developing AKI (AKI). CON denotes sham-operated control groups. Results are expressed as mean ± SEM. # designates *p* < .05 vs. control. HIF1A - Hypoxia inducible factor 1, BAX - BCL2-Associated X Protein, HSP27 - Heat shock protein 27, HSP70 - Heat shock protein 70, HSP90 - Heat shock protein 90, HSPA5 - Heat shock cognate 70 kDa protein, NOS2 - Inducible nitric oxide synthase, TLR2 - Toll like receptor 2, TNF - Tumor necrosis factor alpha

To obtain data on the processes that are uniquely related to the development of AKI, we analyzed the differences in gene expression between the control group, NON-AKI septic animals and septic pigs developing AKI. The expression of all selected genes assessed at 22 h of the experiment did not significantly differ between the control and NON-AKI group. In contrast, several genes that were not significantly altered in the NON-AKI group were markedly changed in AKI animals (Fig. 1). The expression analysis revealed a marked increases in expression for PGHS-2 (14.6-fold, *P* = 0.01), AGTR2 (8.1-fold, *P* = 0.01), CASP3 (5.1-fold, *P* = 0.02) and TLR-4 (2.7-fold, *P* = 0.04). In addition, the development of AKI was characterized by statistically significant down-regulation of PGC-1 alpha (2-fold, *P* = 0.02). The expression of HIF-1A was 3.5-fold higher in AKI group compared to NON-AKI, but not statistically significant (*P* = 0.06). Gene expression of BAX, HSP (27,70,90,A5), NOS2, TLR2 and TNF-α showed no significant change compared to NON-AKI group (Fig. 2).

## Discussion

The relevant question of this exploratory study was whether renal tissue gene expression pattern in animals that develop septic AKI differs from pattern seen in septic subjects without AKI. We combined gene expression analysis with a classic large mammal model of sepsis. Since this modeling allowed us to generate septic animals both with and without AKI, the confounding effects of sepsis that are independent of AKI could be isolated and studied. Hence, a unique feature of this study is that we have not compared only AKI animals and control, healthy animals, as in previous studies in rodent models [4, 5], but extended this analysis to compare the gene expression in septic animals with and without AKI. The facts that AKI is affecting 40–50% of all septic patients [6] and that differences between septic patients with and without AKI are only poorly understood lend strong relevance and usefulness to our approach. In addition, by coupling this approach with traditional parameters of renal physiology, our study helped establish a picture of molecular-based differences between septic subjects with and without AKI. Our preliminary results show that the vulnerability of kidneys to develop AKI in sepsis is associated with early, exaggerated systemic inflammatory response coupled with different trajectories in tissue expression profile of genes representative of inflammatory, metabolic and apoptotic responses compared to AKI free septic animals.

In our study, septic animals that developed AKI had systemic hemodynamic phenotype (normo- or hyperdynamic) comparable to non-AKI subjects, but demonstrated higher systemic inflammatory response, an increase in renal vascular resistance and early fall in cortical microcirculatory blood flow. Our results are in excellent agreement with those from observational clinical studies [7–10]. Indeed, Murugan et al. demonstrated that patients admitted with community acquired pneumonia who presented with or developed AKI had higher plasma levels of IL-6 and TNF-α than non-AKI patients [7]. Likewise, Payen et al. recently showed that the development of septic AKI strongly reflects the intensity of systemic cytokine levels [8]. However, the mechanistic link between systemic inflammation and renal injury remains to be demonstrated. In this context, our study further extends these clinical observations by demonstrating the temporal relationship between exaggerated systemic inflammatory stress, intra-renal hemodynamic alterations and significant changes in kidney gene expression in AKI subjects as opposed to septic subjects without AKI.

Rather than utilizing genome-wide approach, we focused on prospectively selected subset of genes most likely to be implicated in major pathogenic pathways. We observed different intrarenal expression of TLR-4, which was found to be significantly up-regulated in AKI animals only, a finding consistent with previous rodent data [5]. TLR-4 receptors are constitutively expressed at RNA level both on renal endothelial and epithelial tubular cells and represent pattern recognition receptors, which can be activated by both pathogen-associated molecular patterns (PAMPs) and endogenous ligands called damage-associated molecular patterns (DAMPs) [11, 12]. Upon activation by systemic or local danger signals, intrarenal TLR-4 drives local innate immune and inflammatory responses. Our results fit well to an attractive hypothesis that exposure of the endothelial and tubular epithelial cells to such a high amount of circulating cytokines delivered to these cells via both peritubular microcirculation and nephron filtration might trigger TLR-4-dependent propagation of microvascular and tubular injury [13]. Thus, targeting the ligand-TLR-4 interaction may represent a promising therapeutic target to tackle the endothelial and epithelial dysfunction in kidney injury [14–16]. The up-regulation of renal TLR-4 might also be closely linked to the expression of the gene for cyclooxygenase-2 (PGHS-2), which was found up-regulated 14.6-fold compared to NON-AKI group in the current study. Indeed, TLR-4 dependence of cyclooxygenase-2 has been shown in rodent model of cecal ligation and puncture [17]. In the mammalian kidney, cyclooxygenase-2 is predominantly expressed in macula densa, medullary interstitial cells, cortical thick ascending limb and the endothelium of renal arterioles [18, 19]. Cyclooxygenase-2-derived prostanoids appear to play a critical role in regulating renal hemodynamics, tubuloglomerular feedback mechanism and medullary salt and water excretion [18, 19]. Our findings are consistent with previous experiment reporting increases in renocortical cyclooxygenase-2 mRNA and protein expression in a rat model of endotoxemia [20]. Moreover, in that study cyclooxygenase-2 inhibition ameliorated endotoxin-induced renal dysfunction [20]. Therefore, further delineation of downstream effects of cyclooxygenase-mediated renal physiology appears to be justifiable in septic AKI.

Another prominent change was that only animals with septic AKI had profoundly down-regulated expression of PGC-1 alpha, a critical regulator of mitochondrial metabolism and biogenesis. The importance of PGC-1 alfa in the pathogenesis of S-AKI has only recently been illustrated in endotoxemic mice, in which a substantial decline in the kidney expression of PGC-1 alfa was accompanied by biochemical and structural evidence of renal tubular mitochondrial dysfunction [21]. In addition, PGC-1 alpha knockout mice suffered from persistent kidney injury following endotoxemia, suggesting that sustained suppression of PGC-1 alfa induced by inflammatory stressors could play a role in the transition from adaptive alterations in cellular energy metabolism of tubular cells to injurious event in AKI [21]. Thus, the down-regulation *of* PGC-1 alpha described for the first time in our large animal model lends further credence to this concept. Potentially equally important is the finding of increased expression of the pro-apoptosis-related gene caspase-3 in AKI animals, because the putative role of apoptosis in the pathogenesis of AKI still remains controversial [22–24]. Finally, pigs with septic AKI showed a marked increase in the expression of AGTR2. The rationale for looking at AGTR2 gene expression rested on the finding that angiotensin type 2 receptor-dependent pathways seem to participate in renal inflammatory processes and apoptosis in various types of acute renal injury [25].

Understanding the methodological limitations of our experimental approach is essential for any translational research. First, the design of the present study neither allows one to establish a causative link between observed genetic signature and kidney injury nor provide mechanistic insights of factors that regulate specific gene expression. Of note, both massive systemic inflammation and local hypoxia due to intra-renal microvascular derangements might influence renal tissue gene expression pattern. Hence, it is not possible to unambiguously determine the primacy and impact of inflammatory stress, renal circulatory alterations or both on genetic signature. The absence of significant increase in HIF-1A, a master regulator of adaptive cellular responses to hypoxia [26], might suggest that inflammation rather than substantial widespread tissue hypoxia is the primary and predominant event in early phase of septic AKI. Second, due to the small number of subjects, we were not powered to compare the effects of the two types of sepsis models on gene expression. Therefore, we cannot exclude model-specific differences [27]. On the other hand, clear-cut differences seen in a number of biologically relevant genes between AKI positive and AKI negative animals support the validity and usefulness of this pooling approach. Third, renal injury in sepsis is a dynamic process and a single snap shot analysis at one time period provides only limited perspective.

Furthermore, studies using tissue samples of the entire kidney might be confounded by tissue heterogeneity. In complex conditions such as sepsis and AKI alterations in renal genomic profile might be a consequence of changes in intrinsic renal tissue constituted by diverse cellular types and, to a variable degree, by infiltrating inflammatory cells. In addition, the finding of differentially expressed genes might not be unique to the kidneys. In support of this notion, Grigoryev et al. found striking similarity in transcription patterns of inflammatory genes in kidney and lung tissue in a murine model of ischemic AKI [28]. Finally, we studied young, otherwise healthy animals. Several risk factors like aging, hypertension, diabetes and chronic kidney disease are known to significantly increase the susceptibility to AKI in sepsis. Understanding the differences in renal genomic response in the presence of pre-existing comorbidities in comparison to healthy kidneys would be highly informative. Molecular genetic response in septic AKI is multifaceted, and, hence, number of relevant genes might have been missed.

## Conclusion

We report the first attempt to distinguish subjects with septic AKI from those with sepsis alone based upon renal tissue gene expression in a large animal model. Combined gene expression analysis with a classic large mammal model of sepsis including integrative physiologic monitioring and complex intensive care of AKI animals may guide further research. Genes whose changes in expression levels were associated with the development of septic AKI are representative of local inflammatory and danger overstimulation (TLR-4 pathway) and linked to metabolic (PGC-1 alpha) and apoptotic (caspase 3) molecular responses. This genomic profile, albeit independent from systemic hemodynamics, is associated with remarkable systemic inflammation and different intra-renal hemodynamic phenotype compared with sepsis without AKI. Cyclooxygenase-2 derived lipid mediators and angiotensin type 2 receptor-dependent downstream mechanisms appear fruitful targets for future mechanistic studies. Despite the biological plausibility, our experimental data need to be treated as exploratory, thus requiring further validation.

## Abbreviations

AGTR2: Angiotensin II Receptor, Type 2
AKI: Acute kidney injury
BAX: BCL2-Associated X Protein
CASP3: Caspase 3
HIF1alfa: Hypoxia inducible factor 1
HSP 27: Heat shock protein 27
HSP70: Heat shock protein
HSP90AA1: Heat shock protein 90
HSPA5: Heat shock cognate 70 kDa protein
NOS2: Inducible nitric oxide synthase
PGC-1 alpha: Peroxisome Proliferator-Activated Receptor Gamma, Coactivator 1 Alpha
PGHS-2: Cyclooxygenase-2
S-AKI: Sepsis-induced acute kidney injury
TLR-2: Toll like receptor 2
TLR-4: Toll like receptor 4
TNF-α: Tumor necrosis factor alpha
